# DeNO*_x_* Abatement over Sonically Prepared Iron-Substituted Y, USY and MFI Zeolite Catalysts in Lean Exhaust Gas Conditions

**DOI:** 10.3390/nano8010021

**Published:** 2018-01-03

**Authors:** Damian K. Chlebda, Patrycja Stachurska, Roman J. Jędrzejczyk, Łukasz Kuterasiński, Anna Dziedzicka, Sylwia Górecka, Lucjan Chmielarz, Joanna Łojewska, Maciej Sitarz, Przemysław J. Jodłowski

**Affiliations:** 1 Faculty of Chemistry, Jagiellonian University, Gronostajowa 2, 30-387 Kraków, Poland; damian.chlebda@uj.edu.pl (D.K.C.); gorecka.syl@gmail.com (S.G.); chmielar@chemia.uj.edu.pl (L.C.); lojewska@chemia.uj.edu.pl (J.Ł.); 2 Faculty of Chemical Engineering and Technology, Cracow University of Technology, Warszawska 24, 31-155 Kraków, Poland; patrycja.stachurska@gmail.com (P.S.); dziedzicka@chemia.pk.edu.pl (A.D.); 3 Malopolska Centre of Biotechnology, Jagiellonian University, Gronostajowa 7A, 30-387 Kraków, Poland; roman.jedrzejczyk@uj.edu.pl; 4 Jerzy Haber Institute of Catalysis and Surface Chemistry, Polish Academy of Sciences, Niezapominajek 8, 30-239 Kraków, Poland; nckutera@cyf-kr.edu.pl; 5 Faculty of Materials Science and Ceramics, AGH University of Science and Technology, al. Mickiewicza 30, 30-059 Kraków, Poland; msitarz@agh.edu.pl

**Keywords:** deNO*_x_* selective catalytic reduction (SCR), zeolites, sonochemistry, iron oxide, Fe-zeolites

## Abstract

Iron-substituted MFI, Y and USY zeolites prepared by two preparation routes—classical ion exchange and the ultrasound modified ion-exchange method—were characterised by micro-Raman spectroscopy, X-ray diffraction (XRD), scanning electron microscopy (SEM), and ultraviolet (UV)/visible diffuse reflectance spectroscopy (UV/Vis DRS). Ultrasound irradiation, a new technique for the preparation of the metal salt suspension before incorporation to the zeolite structure, was employed. An experimental study of selective catalytic reduction (SCR) of NO with NH_3_ on both iron-substituted reference zeolite catalysts and those prepared through the application of ultrasound conducted during an ion-exchange process is presented. The prepared zeolite catalysts show high activity and selectivity in SCR deNO*_x_* abatement. The MFI-based iron catalysts, especially those prepared via the sonochemical method, revealed superior activity in the deNO*_x_* process, with almost 100% selectivity towards N_2_. The hydrothermal stability test confirmed high stability and activity of MFI-based catalysts in water-rich conditions during the deNO*_x_* reaction at 450 °C.

## 1. Introduction

NO*_x_* compounds (NO and NO_2_) are products of the internal combustion of fuels. The problem of their removal from exhaust gas is crucial, mostly because of their negative environmental impact [1,2,3]. Combustion in diesel vehicles and power plants can be considered to be their main sources. The process is a lean burn, so there is a significant amount of unreacted O_2_ in the exhaust gases. Nowadays, the emission limits for NO*_x_* concern both stationary and mobile sources, and the maximum allowed concentration level in the air has become more restricted in recent years [4,5]. NO*_x_* removal is also important due to the compounds’ behaviour as catalysts for photochemical oxidation, and because N_2_O is classified as one of the greenhouse gases. Commercially, NO*_x_* exhaust gases are neutralised by the application of selective catalytic reduction (SCR) with ammonia used as a reducer [6]. Currently, SCR is the most efficient process for converting NO*_x_* into molecular nitrogen in the presence of oxygen from the lean exhaust. The development of catalytic materials that will be active in this process has to meet several requirements: the structure of the catalyst has to be resistant to high temperatures, exhibit high values of mass and heat transport, and demonstrate high selectivity, especially to N_2_.

Transition metal oxide-based catalysts have been widely used and analysed in deNO*_x_* abatement [7,8]. However, their activity is higher at a lower range of temperatures than other materials currently being investigated. Over the past few years, the interest of researchers has been focused on the application of zeolites and their modification to be used even at elevated temperatures. In the literature, the application of perovskite materials has gained much attention for their potential as new materials for use in the deNO*_x_* process [9]. The preparation of the active catalysts for deNO*_x_* purposes includes several methods that are frequently used: hydrothermal synthesis, solid-state and aqueous exchange, sublimation or chemical vapour, and new methods such as the application of ultrasound irradiation. The selection of a specific method of catalyst preparation may result in different properties of the material, such as acidity, the nature and loading of the incorporated transition metal ion, surface properties, and the available number of active sites [10,11]. Herein, the influence on other metals present in the catalyst should also be considered. The application of ultrasound irradiation for zeolite synthesis allows reduced time and temperature during the preparation, which may result in decreased molecule size and increased crystallinity of the prepared material. In the literature, sonochemically prepared catalysts have recently been applied for the purpose of selective catalytic reduction. In work [12], the Cu-based zeolites exhibited high activity, achieving the maximum conversion together with high selectivity towards the N_2_ compared to the reference materials prepared by the standard ion-exchanged method. The search for even better catalysts for the deNO*_x_* process has resulted in abandonment of the commercially used V_2_O_5_–WO_3_/TiO_2_ and other catalysts, which include carcinogenic active metal [13] and have a negative impact on the environment. The zeolites believed to be the most promising catalysts for NO*_x_* decomposition are mostly based on Cu and Fe as active metals substituted in the zeolite framework. In the literature [4,14], several types of zeolite have been employed including ZSM-5, Y and BETA, and have been optimised to provide high activity and selectivity at both low and high temperatures. Examples of classical synthesis can be found in [14], where two different conventional procedures of ion exchange and impregnation were used to prepare Cu- and Fe-based catalysts supported on BETA and ZSM-5 zeolites. The results showed very high SCR activity of Cu catalysts even at lower temperatures compared to the Fe catalysts. However, as has been noted elsewhere [4], Fe catalysts do not cause the formation of small amounts of nitrous oxide, as was detected over the copper catalyst even with negligible NO_2_ feed content. Vy contrast with what is described in this publication, MFI and Y based zeolites and those containing small-pore zeolites may be considered as alternative ammonia SCR catalysts and, recently, have been exhaustively investigated [15,16]. It is considered easier to understand the activity-structure relationship and introduced metal active sites properties of the small-pore molecular sieves with a Chabazite (CHA) structure such as SSZ-13 and SAPO-34 [17]. For the ZSM-5 and other medium- and large-pore zeolites, some progress on the understanding of iron structure active sites has been made, although the mechanism is still not fully understood [16]. Studies on the Fe/SSZ-13 catalyst proved its good catalytic activity and hydrothermal resistance in deNO*_x_*, however researchers have suggested their use as a co-catalyst to Cu-based ones in order to extend the catalytic activity to lower temperatures [17,18]. Corma et al. compared the activity and resistance of the catalysts against steam for Fe-BETA and Fe-CHA using a different preparation route. For selected examples, the small-channel zeolite catalyst exhibited high and even better properties in ammonia SCR than their counterparts [19]. Despite high activity, some of the zeolite catalysts are not resistant to the presence of inhibitors and poisoning. The inhibition of SCR activity has been described, e.g., for Cu-ZSM-5 catalysts in the presence of oxygen, water or sulphur oxide [20]; some of them even caused irreversible changes in the catalyst’s structure. The observed behaviour of catalysts can be overcome through different catalyst compositions and preparation routes. The addition of the second active metal and a new synthesis route have shown a promising resistance of catalysts to the co-presence of water and sulphur during NO*_x_* decomposition of the Fe-Ce-ZSM-5 catalyst obtained from the new synthesis path [21].

Iron and copper are most often applied as the active metal in the SCR process. Analysis of the iron form is difficult, and it may coexist in different oxidation states in the same catalyst sample. This depends also on the material used as a precursor for ion exchange [22], the preparation method, the activation conditions (temperature, presence of other molecules, etc.) and the Fe loading [23]. Iron is widely distributed, and it can be found in many materials (including zeolite materials) as an impurity. The identification of the active form of iron in zeolite materials is challenging, because there are many possible structures of Fe species in zeolites. However, it is known that monovalent, divalent and trivalent iron ions, as well as several oxo and hydroxo-complexes and many iron oxide forms, may coexist and be present in catalytic material simultaneously [22,23,24]. The examples of iron-substituted zeolite catalytic materials proved that the SCR of NO by NH_3_ is catalysed by different forms of iron molecules. In work [24], the Fe-loaded ZSM-5 catalysts were shown to contain mononuclear Fe sites mainly in the form of Fe*_x_*O*_y_* oligomers that play a significant role in the overall deNO*_x_* process.

Within this study, the main objectives were to characterise the zeolites containing Fe and synthesised using the standard ion-exchange method, and to compare their catalytic properties to the proposed zeolite-based catalysts prepared by the application of ultrasound irradiation during the ion-exchange step. The application of the ultrasound during metal salt solution preparation may lead to further better dispersion of metal species over the catalyst’s surface. The aim was to identify the possible active species and compare the activity of the two series of catalysts in selective catalytic reduction of NO*_x_* with ammonia.

## 2. Results and Discussion

The application of ultrasound radiation has been proposed as a new methodology for the preparation of iron-loaded zeolite catalysts. The zeolites used in this work were synthesised in a laboratory environment in accordance with the well-known route, resulting in three types of zeolites being obtained. These were MFI (named after ZSM-5 (Zeolite Socony Mobil-5)), Y and USY (ultrastable Y). The reference materials were prepared using the classical ion-exchange method to allow comparison of the properties of the sonochemically prepared catalysts with those well described in the literature. The details concerning the Si/Al ratio, naming and other properties of the prepared final catalysts are presented in Table 1. It can be seen from the atomic absorption spectroscopy (AAS) results that the catalysts’ loadings are similar taking into account the similar types of zeolites (MFI with a different Si/Al ratio than both Y and USY zeolites). The amounts of iron in reference catalysts were similar to those prepared sonochemically, but for Fe/MFI/15/s the results showed almost two times less iron content compared to the Fe/MFI/15 catalyst (Table 1). The loading of iron in the zeolite framework for USY catalysts was significantly higher than presented in the literature. In work by Perez-Ramírez et al. [25] the Fe−USY catalysts containing 1.28 wt % Fe were tested in N_2_O decomposition. In the work of Li L.D. et al. [26], the catalytic decomposition of N_2_O to N_2_ and O_2_ was performed over Fe−USY consisting of 3.38 wt % of iron. In work by Kern et al. [27], commercially available Fe–zeolite powder with high Fe content (5.5 wt %) was tested in the SCR NH_3_ deNO*_x_* reaction. In work by Gao et al. [16], the Fe content in Fe/SSZ13 catalysts for SCR NH_3_ deNO*_x_* varied from 0.27–1.20 wt %. Thus, considering the loadings of iron used by other researchers, the prepared catalysts based on Y and USY zeolite can be classified as highly loaded, and contain even more added iron than is seen in the literature, while the MFI-based catalysts consist of an average amount of iron introduced to the framework of the zeolites. Nevertheless, in this study both tested catalysts of each type have almost equal content of iron, thus their activity could be compared.

The results of specific surface areas and total pore volumes (cf. Table 1) determined by nitrogen adsorption did not show any correlation between the sonicated and classical catalysts. For the catalysts with the same type of zeolite, the presented values were similar, with the only exception being for Fe/Y and Fe/Y/s catalysts, wherein the reference material had significant lower specific surface area and total pore-volume values. However, some dependences can be noted when comparing these values with those for the synthesised zeolites before modification.

The specific surface areas of the synthesised zeolites were significantly higher in comparison to the catalyst samples. The specific surface area for the Fe-loaded zeolites decreased and, for example, the Fe/USY sample achieved the values of 374 m^2^/g and 317 m^2^/g for the classical and sonicated preparation paths, respectively. However, before modification the specific surface area reached a value almost twice as high (cf. Table 1 and Table 2). A similar observation can be made when comparing the total pore-volume results. The total pore volume measured for the pure zeolite, for example for USY, was equal to 0.376 cm^3^/g, whereas for the catalyst samples it decreased considerably, to 0.273 and 0.236 cm^3^/g for classical and sonicated catalysts, respectively. An exception may be noted for MFI-based catalysts, for which the differences in described values before and after modification did not vary significantly.

The nature of the active sites in prepared samples was examined by Fourier-transform infrared spectroscopy (FTIR) through in situ sorption studies using NH_3_. NH_3_ chemisorption can yield information about the presence of Brønsted and Lewis active sites. The results of the sorption studies are presented in Table 1. When comparing the concentration of Brønsted sites within the groups of zeolites, there was no significant difference. For USY and Y zeolites, the concentration of Brønsted sites varied between 170–205 μmol/g, whereas for USY and Y zeolites prepared by the sonication method, this value was slightly higher, equal to 177 and 244 μmol/g, respectively. A similar tendency can be observed for MFI-15 zeolites, where the concentration of Brønsted active sites was equal to 293 μmol/g for the catalyst prepared by the ion exchange method, whereas for its counterpart prepared by the sonochemical method a small increase to 324 μmol/g was observed. A considerable increase in the concentration of Brønsted sites could be observed for MFI-37 catalysts. For catalysts prepared using the classical ion-exchange method, the concentration of Brønsted sites was equal to 123 μmol/g, whereas the preparation of catalysts using sonochemical irradiation increases the number of active sites to 263 μmol/g. In an analysis of the concentration of Lewis acid sites (cf. Table 1), no direct correlation with the catalyst preparation can be found. The first assumption for faujasite-based samples (Y and USY) was that, in ultrastabilised samples, Si/Al increases, resulting in a decrease in the number of available ion-exchange positions. However, analysis of the concentration of Lewis acid sites in both the USY and Y catalyst series (Table 1) did not confirm that assumption. The Lewis site concentration was, however, higher in Fe/USY catalysts than in Fe/Y catalysts (cf. Table 1). The origin of that phenomenon can be understood when comparing each specific surface area, which is almost 1.5 times higher for the Fe/USY sample. A similar situation can be observed for the Fe/Y/s sample, in which an increase in the concentration of Lewis sites is correlated with the increase of specific surface area. When comparing the concentration of Lewis sites for the MFI-based catalysts with Si/Al = 15 and 37, the correlation between the number of Lewis acid sites and the number of available exchange position in zeolites is evident. When comparing the USY and Y samples, the increase in the Si/Al ratio results in a three-fold decrease in the concentration of Lewis sites, and the further increase in the Si/Al ratio results in a decrease of the concentration of Lewis sites to ca. 80 μmol/g in both the Fe/MFI/37 and Fe/MFI/37/s catalyst samples.

**Figure 1:**
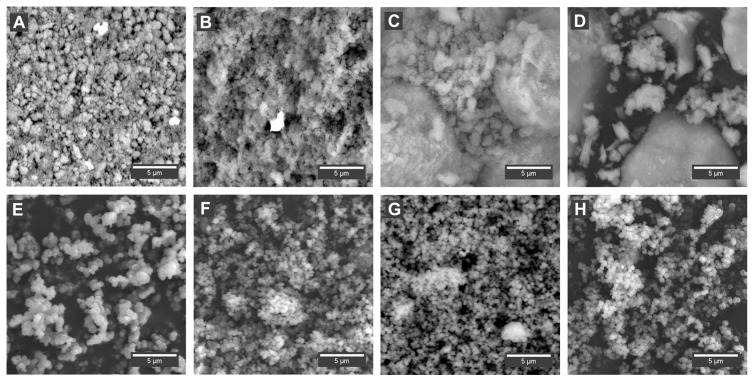
Scanning electron microscopy (SEM) images of iron-based zeolite catalyst samples: (**A**) Fe/MFI/15 catalyst; (**B**) Fe/MFI/15/s catalyst; (**C**) Fe/MFI/37 catalyst; (**D**) Fe/MFI/37/s samples; (**E**) Fe/Y catalyst; (**F**) Fe/Y/s catalyst; (**G**) Fe/USY catalyst; (**H**) Fe/USY/s catalyst.

The morphology of catalysts was examined using scanning electron microscopy (SEM). The images of the surface are presented in Figure 1. The SEM images were taken in backscattered electron mode (BSE), and this measurement methodology allows the differences between the zeolite surface and the deposited active metal particles to be underlined and enclosed. Since zeolites usually have low densities (<2 g/cm^3^) and are made of light elements (Si/Al/O), they are poor electron scatterers. The SEM images do not show the precise distribution of metal particles, but the general shape of the zeolite particles can be observed. The bright spots in the image may indicate the presence of heavier elements over the surface. The MFI-based catalyst particles are of irregular shape, while those of the Y and USY-based catalysts are spherical-like.

**Figure 2:**
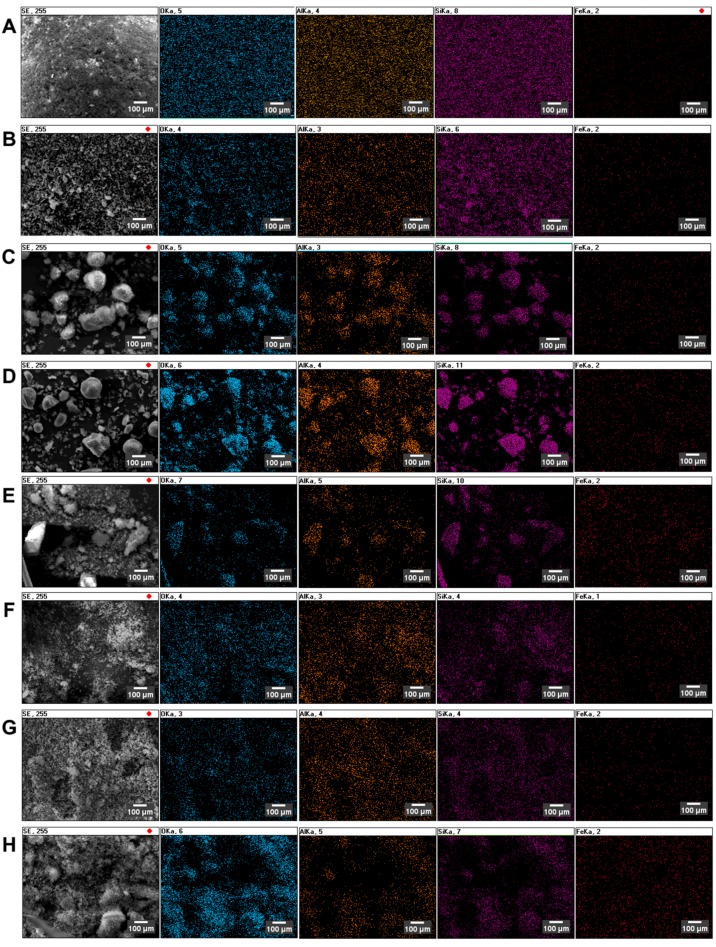
Energy-dispersive X-ray spectroscopy (EDS) distribution maps of selected elements over the surface of iron-substituted zeolites: (**A**) Fe/MFI/15 catalyst; (**B**) Fe/MFI/15/s catalyst; (**C**) Fe/MFI/37 catalyst; (**D**) Fe/MFI/37/s samples; (**E**) Fe/Y catalyst; (**F**) Fe/Y/s catalyst; (**G**) Fe/USY catalyst; (**H**) Fe/USY/s catalyst. Correspondence of colours and elements: O: blue, Al: yellow, Si: magenta, Fe: red.

The determination of the substituted metal distribution was achieved by energy-dispersive X-ray spectroscopy (EDS) mapping, as presented in Figure 2.

Figure 2 represents the spatial distribution of selected elements: O, Al, Si and Fe. The analysis of EDS distribution maps leads to the conclusion that, in all considered cases, the incorporated iron is uniformly distributed over the zeolite. Moreover, there is no correlation between the Fe incorporation method used during the preparation step.

**Figure 3:**
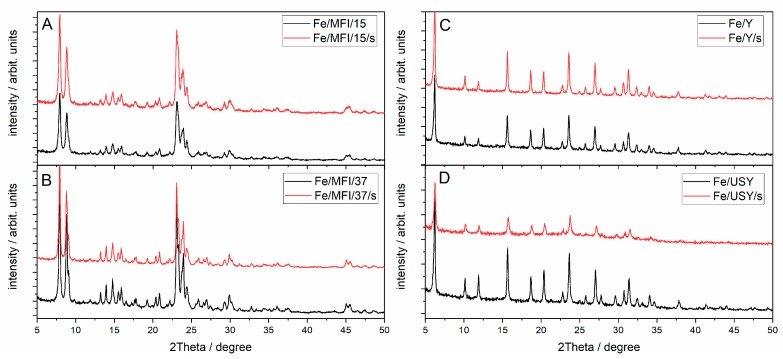
Diffractograms of the iron-substituted zeolites: (**A**) Fe/MFI/15 and Fe/MFI/15/s samples; (**B**) Fe/MFI/37 and Fe/MFI/37/s samples; (**C**) Fe/Y and Fe/Y/s; (**D**) Fe/USY and Fe/USY/s samples.

The X-ray diffraction (XRD) patterns collected for catalysts prepared via the sonochemically aided method and classical ion exchange is presented in Figure 3. The reference diffraction patterns for iron catalysts and pure zeolites are presented in the Supporting Material, in Figure S1. The analysis of the diffraction patterns was performed based on the American Mineralogist Crystal Structure Database [29]. The analysis proved the structures of the zeolites of both sonicated and unsonicated iron-exchanged samples. For Y and USY zeolites before iron exchange, the diffraction peaks are more intense in comparison with the diffraction peaks observed after iron exchange. No XRD patterns of iron oxides or hydroxides were found. This indicates that either the incorporated iron is uniformly distributed within the zeolite structure as cations that exchanged the Al–OH groups, or that there is insufficient amount of iron oxide to give the reflections. In fact, after cation exchange, there is always some fraction of the residual crystallites of oxide or hydroxide type that are unexchanged and occupy the external surfaces of the zeolite grains. This is well described in the literature [26].

**Figure 4:**
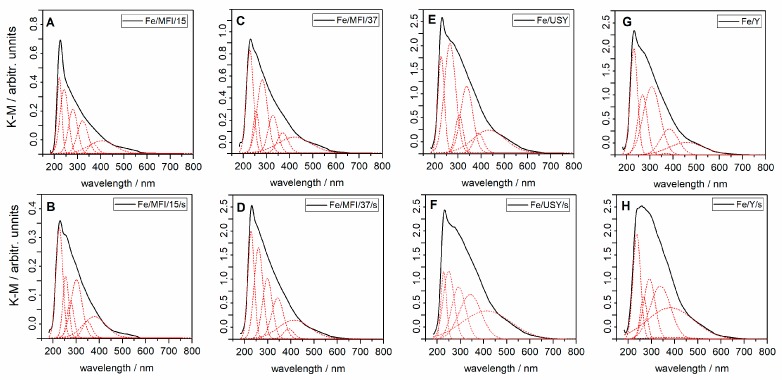
In situ diffuse reflectance UV/visible (UV/Vis) spectra of the iron-substituted zeolites: (**A**) sample Fe/MFI/15; (**B**) sample Fe/MFI/15/s; (**C**) sample Fe/MFI/37; (**D**) sample Fe/MFI/37/s; (**E**) sample Fe/USY; (**F**) sample Fe/USY/s; (**G**) sample Fe/Y; (**H**) sample Fe/Y/s; the red line represents bands calculated in convolution procedure.

Spectral analysis was used to determine the possible coordination of the iron ion within the catalyst samples. The ultraviolet (UV)/visible diffuse reflectance spectroscopy (UV/Vis DRS) spectra of ion-exchanged and sonicated catalysts are presented in Figure 4. The spectra are very broad, so gathering information about the heterogeneous distribution of iron species is not straightforward. The spectra are similar to those found in the literature [26,30,31] for iron-substituted counterpart zeolites. After fitting of the UV/Vis DRS into the lowest possible number of Gaussian sub-bands spectra, several characteristic electron absorption bands can be identified and attributed to the specific iron ion species (in Figure 4, the deconvoluted bands are marked with a red dashed line).

The isolated Fe^3+^ ions in tetrahedral and octahedral coordination can be recognised by bands with centres at about 215 and 280 nm [26]. Discrimination between isolated Fe^3+^ ions in tetrahedral and higher coordination is at present difficult or even impossible, so [32]. UV/Vis spectroscopy is very sensitive to charge-transfer (CT) bands of Fe^3+^, as the position on the band centre depends on the coordination number and degree of aggregation [33]. Three bands for MFI-based zeolites and two bands for Y and USY-based zeolites, with centres between 300 and 400 nm, may indicate the formation of small oligonuclear Fe^3+^*_x_*O*_y_* clusters inside zeolite channels and at the surface, as suggested in the literature [34]. The bands above 400 nm also present on the spectra in Figure 4 can be assigned to Fe^3+^ ions in aggregated form, as observed for Fe_2_O_3_ particles [35]. However, our first assumption was that they are located at the external surface of the zeolite. The above observations are similar for the analysed zeolite catalysts and independent of the preparation pathway. The iron content influences the observed intensity of the spectra characteristics. For high substituted zeolites (USY and Y (compare Table 1)), the absorbance of UV/Vis light is much stronger, as can be seen in Figure 1E–H. The possible Fe^3+^ clusters diffuse into the zeolite channels, and the diffusion for a given zeolite depends on the size of the channels. The identified oligonuclear iron clusters (Fe^3+^*_x_*O*_y_*) in zeolite channels may originate from the condensation on the surface of Al−O−Fe species [26].

**Figure 5:**
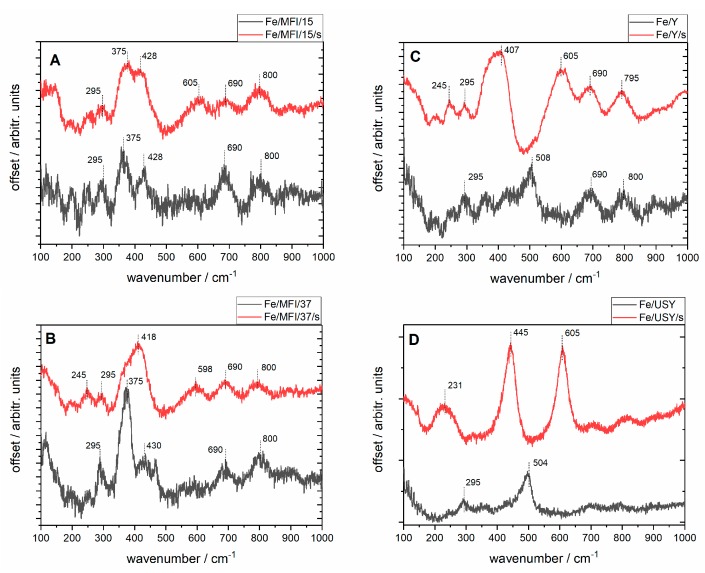
In situ micro-Raman spectra of prepared iron-substituted zeolites registered with 632 nm laser illumination after dehydration in 110 °C in pure helium low; red lines describe the reference spectra of the catalysts prepared with the traditional method, grey lines represent catalysts prepared with the application of ultrasound irradiation. (**A**) Fe/MFI/15 and Fe/MFI/15/s catalysts; (**B**) Fe/MFI/37 and Fe/MFI/37/s catalysts; (**C**) Fe/USY and Fe/USY/s catalysts; (**D**) Fe/Y and Fe/Y/s catalysts.

In situ Raman spectra were registered with a 633 nm laser, as presented in Figure 5. The high level of fluorescence which often occurs when analysing iron-exchanged zeolites was reduced by applying the baseline correction. The common feature of the spectra of the unsonicated samples are the bands at 295, 375, 428, 690 and 800 cm^−1^ except for the USY samples for which the structure is not well resolved. The two bands at 375 and 800 cm^−1^ have been assigned as coming from the zeolites’ network vibrations. The band near 380 cm^−1^ is characteristic of the MFI structure of zeolite (Figure 5A,B), although according to the literature it may be much weaker for modified zeolites (as in this case after Fe loadings) than for pure MFI zeolite [33]. The weak band around 800 cm^−1^ may also come from the symmetric stretching band of the zeolite framework [34]. The bands between 400–500 cm^−1^ observed for most zeolites regardless of preparation route can be attributed to bending modes of Fe−O−O groups. The bands at ~295 cm^−1^ and ~420 cm^−1^ can be assigned to the Fe_2_O_3_ structure, as they can be observed in Raman spectra for iron(III) oxide [35] as the Fe_2_O_3_ molecules may be dispersed on the external surface of the zeolite. Taking this into account, it can be inferred that for the unsonicated USY samples the harsh ultrastabilisation conditions (temperature, water vapour) may have given rise to either an increase in the dispersion or the deterioration of the zeolite structure. It can be noted that for this samples the Fe_2_O_3_ bands are still visible. Additionally, for the unsonicated samples of both Y and USY catalysts, the bands at 508 and 504 cm^−1^ respectively, can be attributed to symmetric stretching/bending vibrational modes of isolated Fe−O−Si bonds [32]. These groups can be described as characteristic of the chemical interaction of extra-framework species with the zeolite siliceous matrix.

Upon sonication, the structure of the samples is modified, which is demonstrated by broadening of the bands and the appearance of the new bands: at 605 cm^−1^ for the Fe/MFI/15/s sample; 418 and 596 cm^−1^ for the Fe/MFI/37/s; 407 and 605 cm^−1^ for the FeY sample; and 231, 445, 605 and 850 cm^−1^ for the Fe/USY/s sample. The bands’ broadening is due to the increase of the samples’ dispersion upon sonication, which is in accordance with the SEM analyses. The band at 605 cm^−1^ is common for all the samples except Fe/MFI/s for which it is shifted to lower frequencies. As mentioned above, since the Fe/USY has a substantially different structure also the vibrational pattern achieved after sonication is different for it, suggesting the formation of the Fe_3_O_4_-dispersed crystallites outside the lattice. Of particular interest for the present study was the observation of different organisation of the surface iron species for USY and Y zeolite, depending on the preparation route of the final catalysts—classical and the sonochemically supported ion-exchange method. The samples with the sonochemical route have more exposed bands attributed to isolated Fe–O–Si bonds’ vibration, while those at around 400–445 cm^−1^ and 605 cm^−1^ are not visible at registered spectra.

**Figure 6:**
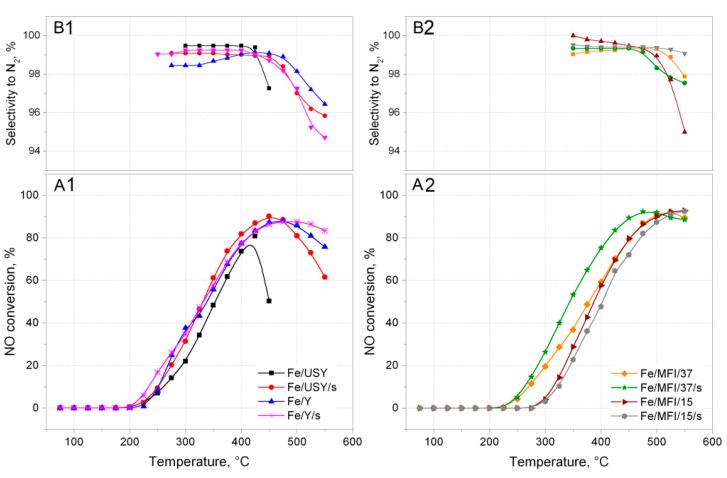
Kinetic results of selective catalytic reduction (SCR) deNO*_x_* over various zeolite catalysts: (**A1**,**A2**) reaction rate; (**B1**,**B2**) N_2_ selectivity in SCR of NO with NH_3_.

The activity of the prepared catalysts in the SCR deNO*_x_* reaction was measured in a plug flow reactor. The results are presented in the form of light-off curves in Figure 6. The activity of catalysts was observed at reaction temperatures of up to 550 °C. It can be noted that all prepared catalyst samples revealed complete NO conversion. It should be assumed that Fe/USY and Fe/USY/s, with their high iron loads and, therefore, with many more oligonuclear clusters present inside the zeolite structure, would exhibit the highest catalytic activity. However, the best activity was obtained by MFI-based catalysts. The MFI/15 and MFI/15/s catalysts can be said to be most active, with no downward trend at 550 °C. Nevertheless, the USY-based catalysts achieved their maximum conversion value at much lower temperatures than the MFI catalysts. Unfortunately, significant deactivation can be noticed for these catalysts above 450 °C, and even faster deactivation for Fe/USY catalysts, above 400 °C. The behaviour of catalyst Fe/MFI/37/s, prepared by the sonochemical preparation route, is interesting. The light-off temperatures and overall conversion are left-shifted to lower temperatures than are those of its counterpart catalyst prepared by the conventional ion-exchange method. It is even more surprisingly that MFI catalysts have over two times less iron introduced into the zeolite than the USY and Y catalysts. However, to comprehensively compare the overall catalytic performance of iron-substituted zeolite catalysts prepared in both ways, selectivity towards N_2_ should also be considered.

Figure 6B1,B2 presents the selectivity curves calculated for each catalyst sample. The selectivity reached high values and varies mostly between 96–99%. However, a selectivity drop can be noted for almost all catalysts above 450 °C (for USY catalysts) and 475 °C (for MFI catalysts). The only exception is the Fe/MFI/37/s catalyst, in which selectivity is almost constant and varied between 99–100% throughout the tested temperature range. The high loading and effective dispersion of Fe-species, leads to high activity, especially in the case of catalysts prepared with ultrasound irradiation. More catalytic active sites are provided by the identified isolated Fe^3+^ ions and oligonuclear iron oxides, and their amount depends on the type of zeolite and preparation route of the catalyst. The activity of the described catalysts is even better than that reported in the literature for the deNO*_x_* process. In publication [26], authors tested three zeolite-based catalysts and reported higher activity of Fe-USY catalysts than Fe-Beta and FeZSM-5 catalysts prepared in the same wet ion-exchange manner, and in publication [31] the authors also tested different zeolites (i.e., USY). The observed light-off curves are slightly right-shifted to the higher temperatures than the results reported in this paper, with worse selectivity parameters.

**Figure 7:**
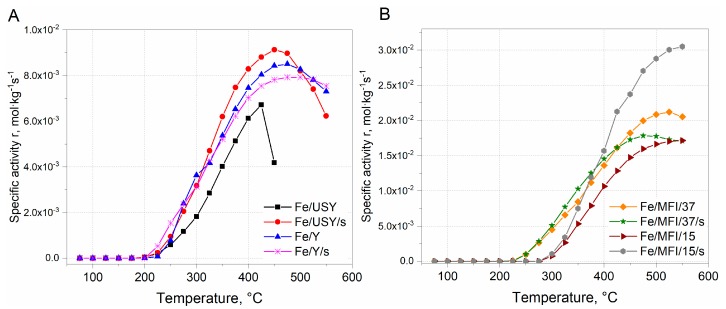
Specific activity of selective catalytic reduction (SCR) deNO*_x_* over various zeolite catalysts: (**A**) USY and Y zeolites; (**B**) MFI/15 and MFI/37 zeolites.

Since, the differences in Fe loading in between USY, Y and ZSM-5 catalysts samples are evident, to compare the activity between the series, the reaction rate was expressed in a form of the specific reaction rate related to the catalysts’ loading. The results are presented in Figure 7.

The comparison of the specific reaction rates within the series of the catalysts are similar to those expressed in Figure 6A1,A2. It can be found that the within the group of USY and Y iron-substituted catalysts, the highest activity was obtained for the Fe/USY/s catalyst sample. The maximum reaction rate was ca. 9 × 10^−2^ mol·kg^−1^s^−1^ at 450 °C (Figure 7A). The activity of other faujasite-based catalysts decreased in the following order: Fe/Y > Fe/Y/s > Fe/USY. However, the comparison specific reaction rate values calculated for MFI-based catalysts shows that the activity of MFI-based catalysts is ca. one order of magnitude higher than that of faujasite-based samples. The maximum activity was obtained by Fe/MFI/15/s catalyst samples and equal to 3.0 × 10^−2^ mol·kg^−1^s^−1^ (Figure 7B). Considerably lower activity was revealed by the Fe/MFI/37 catalyst sample. The lowest activity was obtained by Fe/MFI/37/s and Fe/MFI/15 catalysts. The high activity of Fe/MFI/15/s catalysts is a derivative of the iron content which induces the high number of Lewis acid sites which are active in NH_3_ SCR deNO*_x_* reaction [16]. Indeed, for the MFI-based catalysts with the lowest Fe loading—Fe/MFI/15/s and Fe/MFI/37 catalysts—the reaction rate was the lowest and comparable to faujasite-based samples. However, the comparison should also take into account both selectivity at T_50%_. Iwasaki et al. [36] reported the that the reaction rate of SCR deNO*_x_* on Fe/MFI catalysts prepared by impregnation, reductive solid-state ion exchange and chemical vapour deposition methods are not affected by the type of the preparation method. The turnover frequency (TOF) values in SCR deNO*_x_* (mixture 0.1% NO, 0.1% NH_3_, 8% O_2_, 10% CO_2_, 8% H_2_O) were in a range of 9 × 10^−3^ at 473 K to 2.5 × 10^−2^ at 523 K. When comparing the specific reaction rates, the obtained results may partially confirm the findings by Iwasaki et al. [36].

However, it must also be emphasised that both preparation routes may result in the formation of different forms of iron. Thus, correlation between specific reaction rates has to be related to the Fe form present in prepared catalyst samples. To determine quantitatively the forms of the Fe over the prepared samples, the UV/Vis spectra (Figure 4) were deconvoluted to the individual bands. Semi-quantitative analysis of the Fe form in the prepared samples is presented in Table 3.

No direct correlation between the method of preparation and the presence of the specific Fe species can be found. One could say that the preparation of the catalyst by the ion-exchange method should result in the presence of isolated Fe^3+^ rather than large Fe_2_O_3_ particles. Conversely, all species are likely to occur when the sonochemical route is used. However, the results presented in Table 3 show that, in the case of MFI-based catalysts, isolated Fe^3+^ in tetrahedral and higher coordination is in excess, whereas for faujasite-based catalysts the Fe*_x_*^3+^O*_y_* cluster species located inside zeolite pores are predominant. The origin of this can be traced to the manner of zeolite preparation. The hydrothermal treatment in Y zeolites may result in the formation of mesopores and macropores in the zeolites, which favours the formation of oligomeric Fe*_x_*^3+^O*_y_* clusters [37]. On the other hand, for sonically prepared catalyst samples for both catalyst series, significant amounts of oligomeric Fe*_x_*^3+^O*_y_* clusters can be observed. In the literature [16,38], the high activity of zeolite catalysts is attributed to isolated Fe^3+^ species. Both catalyst series, Y/USY and MFI catalysts, have a comparable amount of Fe^3+^ in tetrahedral coordination; the MFI-based are also rich in large Fe_2_O_3_ particles. In work presented by Mao et al. [39], large Fe particles were attributed to α-Fe_2_O_3_ species, which mainly contribute in ammonia oxidation. However, comparison of the micropore volume of parent zeolites (Table 2) with the modified catalysts shows almost double the decrease in micropore volume in the cases of USY and Y catalysts. At the same time, for MFI-based catalysts, the micropore volume remains almost constant. One could associate the decrease in activity to pore blocking by large Fe_2_O_3_ particles. However, all zeolites used in this study represent 3D structures and diffusion of the reactants may proceed via various pathways within the zeolite structure. On the other hand, in work by Janas et al. [40], the authors determine that the overall activity of the deNO*_x_* reaction is attributable to mononuclearity of the Fe lattice species rather than the coordination number. However, the exact attribution of the active sites being responsible for SCR deNO*_x_* require the same structural and physicochemical properties in all compared samples [40].

Based on the experimental results, type of zeolite structure as well as the method of catalysts preparation have a profound impact on further activity in SCR deNO*_x_* reaction. As it may be observed from activity results (Figure 6 and Figure 7) higher reaction rates are achieved by MFI-structure catalysts than for USY and Y catalysts. In most considered cases better activity and selectivity were exhibited by catalysts prepared with ultrasound irradiation during the ion-exchange step. An extraordinary feature of zeolites is that, due to their developed system of channels and cavities operating in the microscale, they can all be treated as surface because the reacting molecules can penetrate through the channels. 

**Figure 8:**
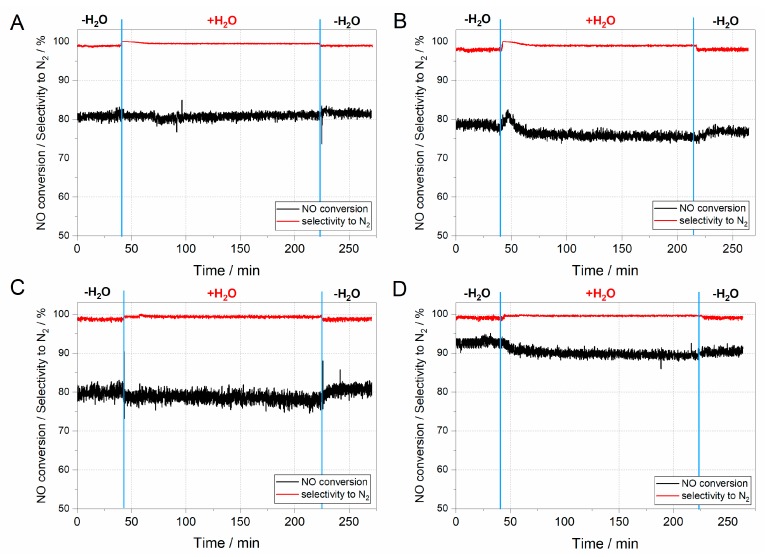
Hydrothermal stability test for ZSM-5 catalysts; (**A**) Fe/MFI/15; (**B**) Fe/MFI/15/s; (**C**) Fe/MFI/37; (**D**) Fe/MFI/37/s catalysts.

Another important feature of the zeolite catalysts for NH_3_ SCR deNO*_x_* is their hydrothermal stability during the overall process. Despite the fact, that very little literature is devoted to the deactivation of zeolite catalysts in NH_3_ SCR deNO*_x_*, the hydrothermal stability of these systems is one of the key issue when considering zeolite catalysts for commercial purposes [41]. The results of hydrothermal stability tests are presented in Figure 8.

The hydrothermal stability tests in all considered ZSM-5 samples started in dry conditions to stabilise the mass spectrometry (MS) signal (Figure 8). After achieving a constant signal, the water vapour was introduced to the mixture of [NO] = [NH_3_] = 0.25%, [O_2_] = 2.5%, [H_2_O] = 5.0% diluted in helium flow. As can be inferred from the results obtained (Figure 8A–D), activity is barely influenced by the introduction of water vapour. Indeed, a slight decrease in activity may be influenced by competitive adsorption of H_2_O at the acidic active centres, which are also the active sites for NH_3_ adsorption [42]. On the other hand, the competitive adsorption of water vapour on the acidic active centres blocks the adsorption centres for ammonia and its further oxidation to NO, which increases the selectivity towards nitrogen.

## 3. Materials and Methods 

### 3.1. Materials

The following materials were used during the synthesis: aluminium nitrate nonahydrate (Chempur, p.a., Piekary Śląskie, Poland), sodium hydroxide (Chempur, p.a.), tetrapropylammonium bromide (TPABr, Sigma Aldrich, 98%, Poznań, Poland), silica (Zeosil, 98%, Gorzów Wlkp., Poland), sodium aluminate (Riedel de Haën, p.a., Seelze, Germany), colloidal silica (Ludox AS-40, 40%, Sigma Aldrich, 98%), iron(III) nitrate nonahydrate (Sigma Aldrich, 98%) and ethanol (Sigma Aldrich, 98%). All chemicals were of analytic reagent grade and were used without further purification.

### 3.2. Zeolite-Based Catalyst Preparation

Within this study, three types of zeolites were synthesised and used to incorporate the active metal inside their structure. The study involves MFI-type zeolite with two Si/Al ratios—15 and 37; Y-type zeolite with Si/Al = 4.52, and ultrastabilised Y (USY) zeolite with Si/Al = 4.52. All types of zeolites were self-prepared and characterised following the procedures presented below.

For the MFI-type zeolite, gels of selected chemical composition were prepared by first preparing two solutions which were further mixed with silica under vigorous stirring and aged for 20 h at ambient conditions. The first solution was obtained by dissolving in an aqueous solution of sodium hydroxide an appropriate amount of aluminium nitrate nonahydrate. The second solution was prepared by the addition of a template—tetrapropylammonium bromide—to the NaOH solution. After the ageing step, the gels were placed into Teflon-lined stainless-steel autoclaves, sealed and aged at 175 °C for 20 h under continuous rotation at 56 rpm. The resulting solids were then centrifuged, washed in distilled water and dried at 80 °C in air-flow conditions. The final step of MFI-type zeolite preparation consisted of the removal of the organic template added during synthesis (TPABr) by high-temperature calcination of the material at 480 °C for 8 h with a temperature ramp of 2 °C/min.

For the Y-type zeolite, the synthesis was performed by adding the colloidal silica under vigorous stirring to the sodium aluminate previously dissolved in the aqueous solution of sodium hydroxide. The obtained gel was then placed into a Teflon-lined stainless-steel autoclave, sealed and kept at room temperature for 24 h. After aging, the autoclave was transferred to the furnace and treated at a temperature of 95 °C for the next 24 h in static conditions. The last step consisted of centrifuging, washing and drying at 80 °C in air-flow conditions.

For the USY-type structure, it is first necessary to ultrastabilise the Y-type structure zeolite. Firstly, the triple ion exchange with a 0.1 M aqueous ammonium nitrate solution at 80 °C for 2 h was performed. After that, ion-exchanged samples were centrifuged and washed three times with distilled water and dried. The obtained ammonium form of zeolite was steamed at 700 °C for 3 h with a temperature ramp of 2 °C/min. Saturated water vapour under 1.25 kPa pressure was applied as a medium with the flow rate equal to 50 mL/min. When the temperature was changed (i.e., during heating and cooling), saturated water vapour was replaced by dry air.

The final catalysts were prepared in two ways: with the standard impregnation (ion-exchange) method and with the impregnation method of ultrasound-treated metal salts solutions. The ultrasound irradiation had been applied to the preparation route, thus the application of classical preparation led to catalysts with large metal-oxide clusters at the surface and the ultrasound resulted in the creation of smaller particles. It is well known that the formation of cavitation bubbles results in the creation of specific high pressure and temperature conditions [43]. During interruptions in the impact of ultrasonic waves on the reaction environment, cavitation bubbles collapse, which is advantageous—the distribution of different size particles is shifted to smaller nm size. The different varieties of prepared catalyst samples are listed in Table 1. Catalysts prepared by the sonochemical method are indicated by the suffix “s”. The reference materials were obtained by immersing the zeolites in a 0.5 M aqueous iron(III) nitrate solution at 20 °C for 24 h. After ion exchange, the samples were centrifuged and washed three times with distilled water and then dried (at 80 °C). Cu-containing zeolites were further calcined in dry air at 500 °C for 4 h with a temperature ramp of 2 °C/min and air flow rate of 50 mL/min.

The sonochemical method involves sonochemical irradiation of zeolites immersed in 0.5 M aqueous iron(III) nitrate solutions and outgassed for 15 min using Ar (Linde, 99.5%, Kraków, Poland) with a flow rate of 20 mL/min. The ultrasound treatment process was performed for 20 min using a QSonica S-4000 sonicator (Church Hill Rd, Newtown, CT, USA) equipped with a ½″ diameter horn. The average power of sonication was 60 W, and frequency was 20 kHz. Directly before sonication, 1.5 mL of ethanol was added to the suspension. The temperature of the suspension was controlled and kept below 60 °C with the use of an ice bath. Further steps of centrifugation, drying and calcination were carried out as described for the reference materials. The final catalyst powder was compressed, crushed, and then sieved to gather grains of 300–600 μm.

### 3.3. Iron Content Analysis

The iron metal content in prepared zeolite-based catalyst samples was determined by atomic absorption spectrometry using a Thermo Scientific ICE3000 series AAS spectrometer (Thermo Fisher Scientific, Waltham, MA, USA). A hollow cathode lamp was used as a radiation source. The external standard method was applied for the determination of metal content (AAS standards, Sigma Aldrich). Signals were processed with Solaar software ver. 2.01 provided by the producer.

### 3.4. Brunauer–Emmett–Teller (BET) Surface-Area Analysis

Brunauer–Emmett–Teller (BET) surface-area determinations were carried out with an ASAP 2000 volumetric adsorption system (Micromeritics Instrument Corp., Norcross, GA, USA). Analysis was based on nitrogen adsorption. The experiments allowed the specific surface area and pore volumes of the prepared catalyst samples to be determined.

### 3.5. X-ray Diffraction (XRD) Analysis of the Catalysts’ Surface

X-ray diffraction data for all prepared catalyst samples was collected using a Panalytical Xpert Pro Diffractometer (Almelo, The Netherlands) equipped with monochromatised Cu Kα radiation (λ = 1.54 Å). Analysis was carried out in the scan range from 5° to 65° 2θ with a scanning step of 0.02° 2θ. In order to determine the framework and extra-framework Al species, XRD measurements of Y and USY samples were carried out according to the procedure described in ASTM D-3942-80 [28]. Knowledge of unit cell sizes allows the use of the Flanigen–Breck equation to calculate the number of Al atoms per unit cell.

### 3.6. Scanning Electron Microscopy (SEM) and Energy Dispersive X-ray (EDS) Analysis

The morphology of the prepared samples was determined by scanning electron microscopy (SEM, FEI Company Nova Nano SEM 200, Hillsboro, OR, USA) in backscattered electron mode. The SEM mapping experiments were performed using a JEOL 5400 scanning microscope (JEOL USA, Inc., Peabody, MA, USA) with a LINK ISIS microprobe analyser (Oxford Instruments, Tubney Woods Abingdon, Oxfordshire, UK). Prior to analysis, the catalyst samples were covered with a carbon layer.

### 3.7. Spectral Properties of the Catalysts Surface

UV/Vis diffuse reflectance spectra were collected by an AvaSpec-ULS3648 High-resolution spectrometer (Avantes BV, Apeldoorn, The Netherlands). Analysis was conducted in an in situ environment by a set-up consisting of a Praying Mantis High-Temperature Reaction Chamber (Harrick Scientific Co., Ossining, NY, USA) with a home-made stainless steel cover equipped with a high-temperature reflection probe (FCR-7UV400-2-ME-H*T*X, 7 × 400 μm fibres, Avantes BV). The sample was illuminated by an AvaLight-D(H)-S Deuterium-Halogen Light Source (Avantes BV) that covers the spectral range of 200–1000 nm. The in situ conditions in which the spectra were registered were provided by a constant flow of pure helium (30 mL/min) at a temperature of 110 °C. These parameters enabled the dehydration of the sample before measurement. For semi-quantitative analysis, the spectra were deconvoluted using OriginPro ver. 9.2 software.

Raman spectra were recorded on a micro-Raman confocal microscope (LabRAM HR, HORIBA Jobin Yvon IBH Ltd., Glasgow, UK) equipped with a deeply depleted thermoelectrically cooled charge-coupled device (CCD) array detector. The slit width was set to 200 mm and the spectral resolution was estimated to be 2.0 cm^−1^. Data were registered using a long working distance lens of 50×. During spectra registration, a 633 nm He-Ne gas laser was used. The procedure of in situ measurements included the precalcination, by heating the sample in a micro-reactor (CCR1000, Linkam Scientific Instruments, Tadworth, Surrey, UK, fitted with quartz windows) to 500 °C in a pure helium flow (30 mL/min) and then cooling to a stationary 110 °C. The spectra were collected in dehydrated conditions at 110 °C in a constant helium flow (30 mL/min).

The concentrations of Brønsted and Lewis sites were determined by in situ FTIR sorption studies. Prior to the sorption studies, the catalyst powders were pressed into self-supporting wafers and activated under vacuum conditions. The catalysts were evacuated at room temperature, then the temperature was increased to 450 °C with a temperature ramp of 5 °C/min until the target temperature was reached, whereby it was maintained for 1 h. After activation, the catalyst samples were cooled and the reference spectrum was recorded. The CO and NH_3_ (Air Products, 99.95%) were distilled by freeze/thaw cycles before adsorption to remove any traces of moisture and impurities. The CO and NH_3_ sorption studies were performed in an IR quartz cell with a calibrated dosing bulb. The amount of probe molecules introduced into the IR cell was calculated from the ideal gas law.

### 3.8. Catalytic Activity Tests

The measurements of the activity of prepared catalysts in selective catalytic reduction of NO with NH_3_ were performed under atmospheric pressure in a fixed-bed quartz microreactor system coupled with a quadrupole mass spectrometer (Prevac, Rogów, Poland). For every sample tested, a standard mass of 0.100 g of catalyst (previously fractioned to particles sizes of 300−600 μm) was weighed and then placed on a quartz wool plug inside the reactor. The tested temperature for the SCR process ranged from 50 °C to 550 °C. The measurement methodology utilised several steps: outgassing the sample in a flow of pure helium at 550 °C for 1 h; cooling the reactor to a temperature of 50 °C; and the SCR process—introducing the gas mixture containing 2500 ppm of NO, 2500 ppm of NH_3_ and 25,000 ppm of O_2_ balanced by helium, with a total flow rate of 40 mL/min and observing the activity of catalysts in a selected temperature range. The registered *m*/*z* during the temperature ramp included: 28 (N_2_), 30 (NO), 44 (N_2_O) and 46 (NO_2_).

### 3.9. Hydrothermal Stability of Prepared Catalysts Samples

The influence of H_2_O on the efficiency of the deNO*_x_* reaction was studied for the ZSM-5 based catalysts by the periodic addition of water vapour into the reaction mixture (5.0 vol %). Helium (used as a balance gas) was switched, by means of a 4-port valve, from dry to wet conditions. At the beginning, the reaction was carried out in a dry reaction mixture ([NO] = [NH_3_] = 0.25%, [O_2_] = 2.5% diluted in helium) for about 45 min. Then, water vapour was introduced into the reaction mixture ([NO] = [NH_3_] = 0.25%, [O_2_] = 2.5%, [H_2_O] = 5.0% diluted in helium) and the test with a wet reaction mixture was conducted for about 3 h. Finally, the wet reaction mixture was switched to a dry reaction mixture and the catalytic test was performed for the next 45 min. All catalytic tests were performed at 450 °C.

## 4. Conclusions

The aim of this study was to characterise and compare the performance of iron-loaded zeolite catalysts in the deNO*_x_* SCR process. Zeolites Y, USY and MFI were used as supports for the ion-exchange with iron, prepared with classical and sonochemical routes. Both methods led to decreased porosity and specific surface area of the catalysts. Differences were found between the Fe loading, depending on the zeolite supports used. USY and Y catalysts have more iron content (even more, in fact, than reported in the literature). UV/Vis and Raman studies indicated that several iron species may be identified as incorporated in the zeolite structure. The isolated Fe^3+^ ions and oligonuclear iron oxides clusters may be identified as active catalytic sites. The prepared catalysts revealed extraordinary activity in SCR deNO*_x_*, although slight deactivation was observed for the USY and Y-based samples. The Fe/MFI/37/s seems to be very promising. This catalyst exhibited superior activity in the deNO*_x_* reaction without deactivation and with almost constant 100% selectivity towards N_2_. Additionally, hydrothermal stability tests have confirmed the high stability of MFI-based catalysts in water-rich conditions.

## Figures and Tables

**Table 1:** Catalysts preparation and characterisation details.

Catalyst Name	Preparation Method	Si/Al	Iron Content *, wt %	*S*_BET_, (m^2^/g)	*V_p_* Total, (cm^3^/g)	*V_p_* Micro **, (cm^3^/g)	Brønsted Sites Concentration, (μmol/g)	Lewis Sites Concentration, (μmol/g)
Fe/USY	Ion-exchange	4.52	8.20 ± 0.41	374	0.273	0.126	170	498
Fe/USY/s	Sonication	4.52	6.73 ± 0.34	311	0.236	0.085	177	519
Fe/Y	Ion-exchange	4.52	7.07 ± 0.35	274	0.229	0.083	205	395
Fe/Y/s	Sonication	4.52	7.54 ± 0.38	448	0.323	0.160	244	566
Fe/MFI/15	Ion-exchange	15	3.69 ± 0.18	307	0.270	0.111	293	164
Fe/MFI/15/s	Sonication	15	2.07 ± 0.10	291	0.259	0.108	324	144
Fe/MFI/37	Ion-exchange	37	2.97 ± 0.15	321	0.255	0.096	123	80
Fe/MFI/37/s	Sonication	37	3.53 ± 0.18	337	0.236	0.103	263	79

* Determined by atomic absorption spectrometry; ** determined from t-plot.

**Table 2:** Parameters of the synthesised Y, USY and MFI zeolites.

Zeolite Name	Si/Al	*S*_BET_, (m^2^/g)	*V_p_* Total, (cm^3^/g)	*V_p_* Micro *, (cm^3^/g)	Brønsted Sites Concentration, (μmol/g)	Lewis Sites Concentration, (μmol/g)
Y	4.52	516	0.353	0.261	241	527
USY	4.52(5.96)	552	0.376	0.246	33	249
MFI	15	297	0.246	0.124	534	88
MFI	37	328	0.219	0.124	435	31

* Determined from t-plot, in case of USY zeolite Si/Al was calculated according to ASTM D-3942-80 [28].

**Table 3:** Numerical analysis of UV/Vis DRS spectra of the catalysts. Percentage of the sub-bands: *I*_1_—band maximum below 300 nm, *I*_2_—band maximum between 300 nm and 400 nm, *I*_3_—band maximum above 400 nm and corresponding wt % Fe of the recognized species.

Catalyst	Fe *, (<300 nm)		Fe **, (300–400 nm)		Fe ***, (>400 nm)		Total Fe, wt %
	*I*_1_, %	wt %	*I*_2_, %	wt %	*I*_3_, %	wt %	
Fe/MFI/15	63.0	2.32 ± 0.11	21.1	0.78 ± 0.04	16.0	0.59 ± 0.03	3.69 ± 0.18
Fe/MFI/15/s	51.5	1.07 ± 0.05	30.2	0.62 ± 0.03	18.4	0.38 ± 0.02	2.07 ± 0.10
Fe/MFI/37	61.3	1.82 ± 0.09	20.6	0.61 ± 0.03	18.1	0.54 ± 0.03	2.97 ± 0.15
Fe/MFI/37/s	46.0	1.62 ± 0.08	33.2	1.17 ± 0.06	20.8	0.74 ± 0.04	3.53 ± 0.18
Fe/Y	39.6	2.80 ± 0.14	46.9	3.32 ± 0.16	13.5	0.95 ± 0.05	7.07 ± 0.35
Fe/Y/s	24.6	1.85 ± 0.09	40.4	3.04 ± 0.15	35.1	2.64 ± 0.13	7.54 ± 0.38
Fe/USY	47.8	3.92 ± 0.20	33.3	2.73 ± 0.14	18.8	1.54 ± 0.08	8.20 ± 0.41
Fe/USY/s	26.2	1.76 ± 0.09	40.9	2.75 ± 0.14	32.9	2.21 ± 0.11	6.73 ± 0.34

* Fe species that corresponds to isolated Fe^3+^ in tetrahedral and higher coordination; ** Fe species that corresponds to oligomeric Fe*_x_*^3+^O*_y_* clusters inside zeolite pores; *** Fe species that corresponds to large Fe_2_O_3_ particles, in the nm range.

## Supplementary Materials

The following are available online at www.mdpi.com/2079-4991/8/1/21/s1, Figure S1: Diffractograms of the iron-substituted zeolites and pure synthesised zeolites: (A) MFI/15, Fe/MFI/15 and Fe/MFI/15/s samples; (B) MFI/37, Fe/MFI/37 and Fe/MFI/37/s samples; (C) Y, Fe/Y and Fe/Y/s; (D) USY, Fe/USY and Fe/USY/s samples.
