# Supplementary material for: DeNOx Abatement over Sonically Prepared Iron-Substituted Y, USY and MFI Zeolite Catalysts in Lean Exhaust Gas Conditions
Source: Nanomaterials (Basel). 2018 Jan 3;8(1):21. doi: 10.3390/nano8010021 (PMC5791108; doi:10.3390/nano8010021)
Supplement: Supplementary file 1 [file nanomaterials-08-00021-s001.pdf]

nanomaterials

Supplementary material

Denox Abatement over Sonically Prepared Iron Substituted Y, USY and MFI Zeolite Catalysts in Lean Exhaust Gas Conditions

Damian K. Chlebda [1], Patrycja Stachurska [2], Roman J. Jędrzejczyk [3], Łukasz Kuterasiński [4], Anna Dziedzicka [2], Sylwia Górecka [1], Lucjan Chmielarz [1], Joanna Łojewska [1], Maciej Sitarz [5] and Przemysław J. Jodłowski [2,*]

[1] Faculty of Chemistry, Jagiellonian University, Gronostajowa 2, 30-387 Kraków, Poland; damian.chlebda@uj.edu.pl (D.K.C.); gorecka.syl@gmail.com (S.G.); chmielar@chemia.uj.edu.pl (L.C.); lojewska@chemia.uj.edu.pl (J.Ł.)
[2] Faculty of Chemical Engineering and Technology, Cracow University of Technology, Warszawska 24, 31-155 Kraków, Poland; patrycja.stachurska@gmail.com (P.S.); dziedzicka@chemia.pk.edu.pl (A.D.)
[3] Malopolska Centre of Biotechnology, Jagiellonian University, Gronostajowa 7A, 30-387 Kraków, Poland; roman.jedrzejczyk@uj.edu.pl
[4] Jerzy Haber Institute of Catalysis and Surface Chemistry, Polish Academy of Sciences, Niezapominajek 8, 30-239 Kraków, Poland; nckutera@cyf-kr.edu.pl
[5] Faculty of Materials Science and Ceramics, AGH University of Science and Technology, al. Mickiewicza 30, 30-059 Kraków, Poland; msitarz@agh.edu.pl
* Correspondence: jodlowski@chemia.pk.edu.pl; Tel.: +48-12-628-27-60

X-ray diffraction (XRD) Analysis

The compassion of intensities of acquired XRD patterns for prepared catalysts samples and pure zeolites is presented in Figure S1.

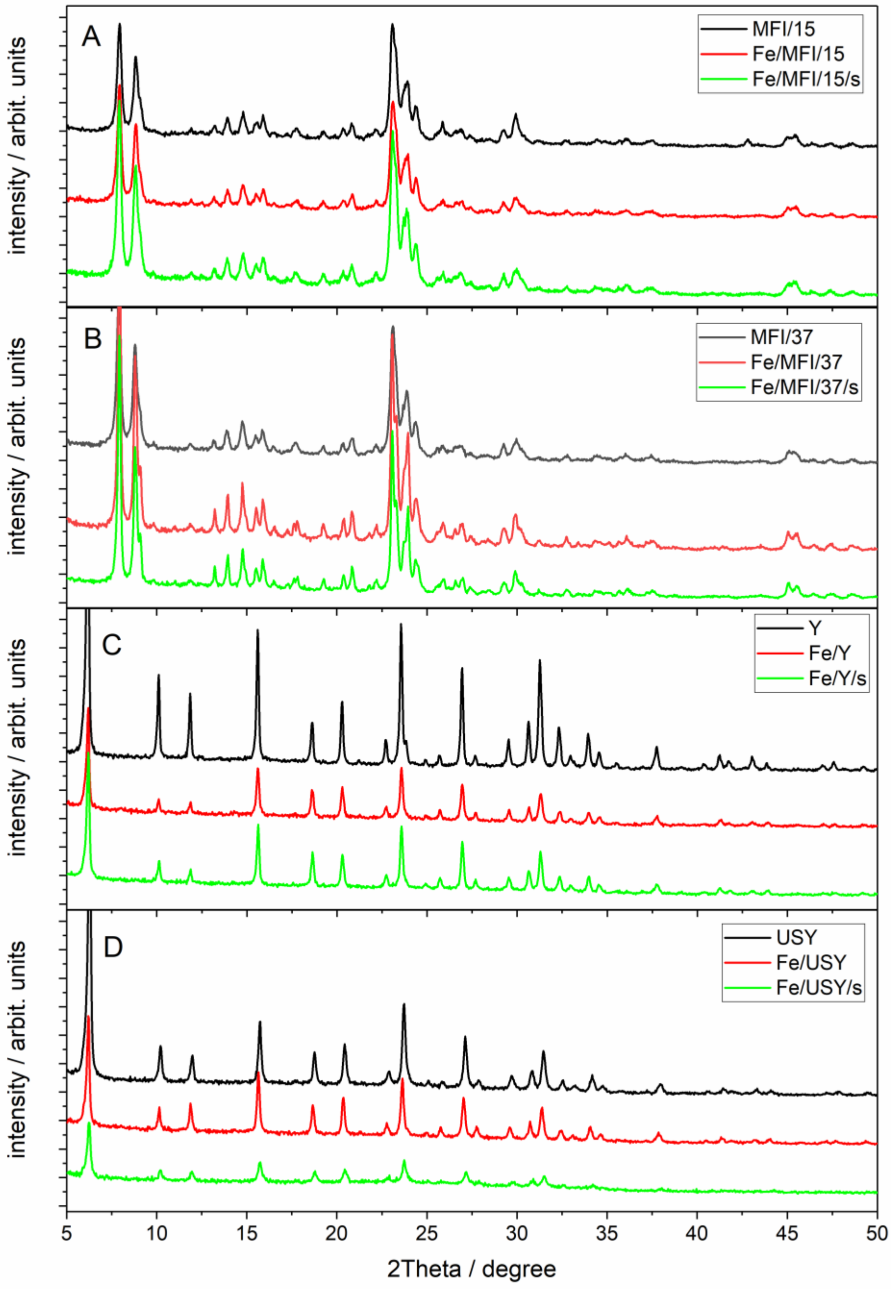

Figure S1. Diffractograms of the iron substituted zeolites and pure synthetized zeolites: (**A**) MFI/15, Fe/MFI/15 and Fe/MFI/15/s samples; (**B**) MFI/37, Fe/MFI/37 and Fe/MFI/37/s samples; (**C**) Y, Fe/Y and Fe/Y/s; (**D**) USY, Fe/USY and Fe/USY/s samples
